# The William Budd Health Centre

**Published:** 1952-10

**Authors:** 


### THE WILLIAM BUDD HEALTH CENTRE

Bristol's new Health Centre was officially opened by the Minister of Health, the Rt. Hon. Ian MacLeod, M.P., on the 16th September 1952 in the presence of the Lord Mayor, members of the City Council and a number of invited guests.

As has been pointed out in a previous article in the Journal by Dr. Wofinden, Deputy M.O. for Bristol, this is the first Health Centre to be built by a local authority for the use of general practitioners. It attempts to solve the problem of inadequate general medical services in a housing area with a population of 30,000. The opening of the Centre brings to an end a long controversy which has gone on in the local press and within the profession, and became *the medical problem of Knowle West.* Our planners in their wisdom erected thousands of new houses on the estate, and while they made provision for a public house and a picture house, no provision was made for a doctor's house or surgeries of any kind. When some of the doctors found it necessary to follow their patients into the estate, they were compelled to use the sitting-room of one of the council houses as a surgery, and the front garden as a very inadequate waiting-room.

The new Centre, which will be used by six general practitioners and will also provide services under the Local Health Authority, does not aim at being any more than a series of suites for general practitioners with a room for minor surgery and a small recovery-room. The Minister, in opening the Centre, pointed out that it was undesirable that precipitate action should be taken in the provision of health centres throughout the country; better to build a few centres of different design and scope to find out by trial and error what was best for the community and the profession. He thought that the present Centre had a great defect in not providing dental and pharmaceutical services. Its advantages, as he saw them, would be that doctors would have more time for the essential work of healing and that they would be able to co-operate more closely with Health Visitors, Midwives, and Officers of the Local Authority.

The Minister also said that he would follow this experiment with keen interest as he was anxious to see the reaction of the patient as well as of the profession. He thought that it would be interesting to see whether the patient preferred to attend a Centre, where the doctor was probably less burdened with work which he could delegate to others, and so could give more individual time to each case, or whether the institutional atmosphere and the herding together of greater numbers would make the patient feel that general practice had lost something of the personal touch. Perhaps in the end he would prefer the old-fashioned doctor with his own surgery.

It must be remembered that the new Health Centre does not provide any diagnostic facilities which doctors do not already possess in their own surgeries. The same number of patients will have to be referred to hospital for blood counts, X-rays, and other investigations which might well be carried out on the spot if the Centre had the necessary equipment and trained personnel. All that has been done is to provide consulting- and waiting-rooms at a capital cost to the ratepayer of £16,000, and a working cost of £10,000 per annum with a return in income of only £400 per annum. It would need thirty or forty such centres to cover the population of Bristol. Is it fair that the rates should be thus burdened, if nothing more is done to provide the general practitioner with laboratory and other services, and take some of the burden from the Hospital out-patients' department-?

The Chairman of the Health Committee, the Rev. Mervyn Stockwood, in introducing the Minister raised a problem which must concern the profession in future. One of the dangers to-day is for doctors to become mere technicians, and for something of the art of medicine to be lost. If health centres become fashionable, the tendency may be for the general practitioner to become like his more privileged colleague, the consultant, a visitor rather than a resident in the area in which he practises. He will have his hours of service; he will see the patients in their homes; but he will only be there during his working hours, and will live away from his practice. Is this a desirable state of affairs? Should not a family doctor belong and live where he does his work, and enter into the life of the community outside his working hours?

Mr. John Wright, F.R.C.S., grandson of Dr. William Budd, a pioneer in Public Health, after whom the Centre has been named, seconded the vote of thanks to the Minister. He took up the point the Minister had made about doctors having more time for essential work, and hoped that the provision of clerical and other facilities would mean less waste of medical men's time. He laid stress on the desirability of trained persons, under the direction of the doctor, dealing with much of the minor illness which now clutters up the surgery of every general practitioner. It will be interesting to see if the doctors working in the new Health Centre will be prepared to pass on work of this nature to assistants if they are available.

It would be fair to say that the ship was launched with hope rather than confidence, and that some of her well-wishers had not a few misgivings about her.

### WILLIAM BUDD

*William Budd* (1811–80) *practised at North Tawton, Devon, as Assistant to his father. He removed to Bristol in* 1842 *and was appointed Physician to St. Peter's Hospital. In* 1847 *he was appointed Physician to Bristol Royal Infirmary.*

*He studied typhoid fever at North Tawton and later in Bristol and his observations were published in the* Lancet *and* British Medical Journal *over a period of more than thirty years. These were reprinted in volume form in* 1873 *entitled "Typhoid Fever, Its Nature, Mode of Spreading and Prevention".*

*His studies included the cause and spread of Cholera and in* 1849 *he published a pamphlet entitled "Malignant Cholera; its Mode of Propagation and its Prevention".*

